# AI-Enhanced Automatic Life Story Structuring for Reminiscence Therapy in Older Adults: Technical Feasibility Study

**DOI:** 10.2196/83122

**Published:** 2026-04-06

**Authors:** Fang Gui, Mengchen Yang, Liuqi Jin, Jing Qian

**Affiliations:** 1 School of Artificial Intelligence, Chuzhou University, No. 1 Huifeng West Road, Chuzhou, Anhui Province, China, 86 13692166474; 2 School of Computer Science and Information Engineering, Hefei University of Technology, Hefei, China

**Keywords:** storytelling, life story organization, caregiver support, Story Mosaic system, older adult well-being interventions

## Abstract

**Background:**

Storytelling interventions have demonstrated substantial potential in improving emotional well-being, cognitive function, and quality of life for older adults. However, its effectiveness is often limited by the challenges of processing disorganized and redundant life stories, which impose substantial cognitive demands on caregivers. Although storytelling interventions are a well-established therapeutic approach, current practices depend heavily on manual narrative organization, restricting both the scalability and consistency of treatment delivery. Prior research has primarily focused on validating the clinical outcomes of storytelling interventions, with insufficient attention given to technological solutions that could enhance narrative processing while preserving therapeutic integrity. Digital approaches to life story structuring remain underexplored, despite their potential to amplify storytelling benefits by reducing cognitive load and improving recall accuracy.

**Objective:**

This study aims to design an event timeline generation algorithm to optimize the prior work of the Story Mosaic system. The optimized system enables (1) the automatic extraction of event elements from life narratives, (2) the automatic organization of fragmented life stories into structured timelines, and (3) the preservation of clinically relevant contextual details during compression. The goal is to reduce manual intervention costs while increasing treatment efficacy through artificial intelligence–driven narrative structuring.

**Methods:**

We have designed a novel method, CARE event timeline (CARE-ET), which combines a temporal attention mechanism with graph-based event relationship modeling. Furthermore, we used the CARE-ET algorithm to optimize existing story collage systems. The system uses multifeature extraction technology to capture event clues from oral histories, prioritizes the 6 elements of events through a hierarchical attention mechanism, and uses adaptive compression algorithms to reduce redundancy while maintaining narrative continuity. To verify the effectiveness of the CARE-ET method, this paper adopts a multidimensional evaluation framework, which encompasses event summary assessment, timeline quality evaluation, and usability testing of the optimized system.

**Results:**

The proposed CARE-ET algorithm outperforms the baseline in both narrative flow and temporal accuracy. The Story Mosaic system, optimized by the CARE-ET algorithm, underwent usability evaluation by 10 caregivers recruited for this study. Based on standardized assessment metrics, the system received an A rating for usability. The comprehensive experimental results demonstrate that the CARE-ET method can effectively structure fragmented narratives from older adults, enhancing the usability of the Story Mosaic system.

**Conclusions:**

The proposed method enables the structured extraction of representative event summaries, transforming disorganized life stories into an event timeline for caregiver-supported older adult well-being interventions. Future research should investigate longitudinal effects on cognitive preservation and explore integration with existing dementia care protocols. This work establishes a critical foundation for intelligent assistive technologies in geriatric mental health interventions.

## Introduction

Cognitive impairment is a common condition affecting the health of older adults, and it is difficult to treat through medication [1]. People with mild cognitive impairment may encounter the following issues: (1) impaired short-term and long-term memory with reduced recall efficiency [2]; (2) deteriorated social functioning, exhibiting social withdrawal and diminished emotional interaction, which can lead to social isolation [3, 4]; and (3) declined executive function in daily activities [5,6].

Through the analysis of over a hundred studies, Zhang et al [7] demonstrated that digital health technologies can effectively address the limitations of traditional medical models by delivering personalized and highly accessible intervention strategies, thereby facilitating timely intervention during the early stages of cognitive decline. Storytelling is an important application of digital health technology, which plays a significant role in the field of reminiscence [8]. Harnessing the power of storytelling, people with mild cognitive impairment can be encouraged to participate more proactively in their health care [9]. Additionally, life stories can serve as a database and decision support tool for understanding older adults and providing personalized services [10-13]. In practice, Zhu et al [14] have demonstrated that storytelling requires older adults to recall, organize, and narrate personal experiences—a process that naturally exercises cognitive functions such as memory, attention, and language abilities.

Interviews and daily interactions with older adults are a common way to access their life stories [15]. However, these life stories often have problems such as redundant content and disorganized structure, which increase the cognitive load on caregivers when learning them [16]. Moreover, as the number of exchanges grows and the volume of life stories continues to expand, the scattered information becomes increasingly difficult for caregivers to process [17,18]. In our prior work, our team developed the Story Mosaic system, designed to assist caregivers in integrating and organizing life stories for older adults. The findings were published in the international journal *JMIR Aging*. However, this version of the system has significant limitations in functional implementation: the extraction of story elements and the arrangement of narrative logic remain highly dependent on manual operations. This not only substantially increases the workload for caregivers but also necessitates the assistance of volunteers in practical applications to complete the recording and organization of older adults’ life stories [17].

An effective solution is to extract the core events from older adults’ life stories, rearrange the events in the order in which they occurred, and generate an event timeline structure of older adults’ life stories. Then, based on the algorithm, develop a visualization system to present the timeline of older adults. An event timeline is a series of events, activities, or important moments recorded and displayed in chronological order, with a simple and clear presentation that allows users to quickly understand the temporal relationship and development of events. The structuring of life stories lies at the critical intersection point between the basic data infrastructure and artificial intelligence applications. It addresses the current problem of “fragmented data and lack of depth” in personalized intervention research, laying the foundation for the development of an intelligent intervention system that truly “understands you” [19].

Existing studies have made some progress in personal event timeline generation, but there are still limitations that do not meet the requirements of older adults’ life stories. The Twitter (subsequently rebranded X) event extraction method proposed by Li et al [20] treats frequent mentions of events as general events, which contradicts the characteristics of older adults’ narratives. Althoff et al’s [21] timeline construction method relies on precise temporal information, which is difficult to apply to the temporally ambiguous life stories. Sun et al’s [22] stage-by-stage story line generation method ensures plot integrity but lacks a generalized description of key events. The pretraining with extracted gap-sentences for abstractive summarization model learns to generate summaries by pretraining with masked key sentences, but its reliance on document structural integrity and sentence coherence makes it difficult to handle issues such as temporal confusion, information redundancy, or fragmentation in the life stories of older adults [23]. The long text-to-text transfer transformer model uses a local-global attention mechanism to efficiently process long texts and avoid excessive information repetition, but its limited ability to capture fine-grained temporal relationships constrains its performance in strictly reorganizing fragmented life events into chronological timelines [24]. The pyramid-based masked sentence pretraining for multidocument summarization model enhances multidocument information integration through pyramid-based masked pretraining; however, as the life stories of older adults are often fragmented and narrated individually, the lack of cross-document structural associations limits the effectiveness of its hierarchical modeling approach [25]. The life stories of older adults exhibit intrinsic particularities, such as ambiguous temporal information, conflicting event elements, and difficulty in determining event significance. These characteristics directly contribute to the limitations of existing methods in processing such narratives.

To address limitations in existing approaches to organizing life stories for older adults, this paper proposes a CARE event timeline (CARE-ET), an innovative event timeline generation method that systematically structures narratives through optimized event relevance analysis and implements the practical Story Mosaic system for caregiver application. Our research makes the following three key contributions. First, we developed a multimodal event fusion method using a 2-layer encoder-decoder architecture that integrates event triggers and timestamps. This structured representation model significantly improves accuracy in extracting key events from older adult narratives. Second, the theoretical novelty lies in the synergistic integration of graph attention networks (GATs) and deep belief networks (DBNs). In contrast to existing hybrids where DBNs serve merely as static feature extractors, the CARE-ET model uses a joint self-supervised module. Here, the latent clustering probability distribution generated by the DBN acts as a global before regularizing the GAT attention coefficients. This ensures that the learned values are optimized not only for local semantic alignment but also for global thematic coherence across the life story timeline. Finally, building upon the CARE-ET method, we have optimized and enhanced the previously developed Story Mosaic system. The improved system is designed to construct more logically coherent life story timelines for older adults, thereby providing reliable data support for personalized care.

## Methods

This section first elaborates on the overall algorithmic framework of the CARE-ET method and concludes with an introduction to the optimized and enhanced functionalities of the Story Mosaic system.

### Ethical Considerations

This study aims to propose a novel algorithm to optimize the Story Mosaic system, thereby better facilitating the automatic organization of fragmented life stories of older adults. This study has been approved by the research ethics committees of Hefei University of Technology (HFUT20220921001) and Chuzhou University (CZSC2025-021), respectively. All participants, including older adults and caregivers, signed a written informed consent form before the initiation of the study. The informed consent form explicitly included authorization for audio recording of the interviews, verbatim transcription of the recorded content, and the use of such materials for academic research purposes. Participation is entirely voluntary. Participants have the right to withdraw unconditionally at any stage of the study without assuming any liability or providing a reason. Given the nature and objectives of this study, there are no circumstances that necessitate the provision of financial or any other form of compensation to participants. All data collected during the research process (including interview recordings and transcribed texts) have undergone rigorous anonymization. All personally identifiable information has been removed or replaced, and comprehensive privacy protection measures have been implemented. The relevant information and images related to the older adults presented in this paper do not involve any personal privacy.

### CARE-ET Method

#### Overview

**Figure 1.**
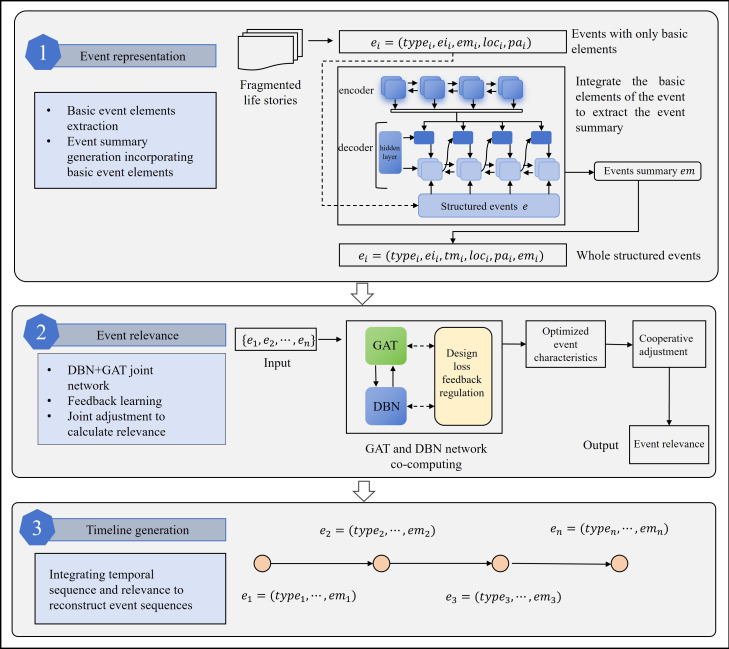
The CARE event timeline (CARE-ET) algorithmic framework for automated life story structuring. The framework comprises three stages: (1) event extraction via bidirectional long short-term memory (BiLSTM), (2) relevance modeling using the joint network of graph attention network (GAT) and deep belief network (DBN), and (3) timeline generation. Rectangles represent computational modules, while arrows indicate the flow of feature vectors and processed narrative data.

The CARE-ET method automatically generates life story timelines through a three-stage computational process, as shown in Figure 1: (1) bidirectional long short-term memory (BiLSTM)-based event extraction and representation, which synthesizes triggers, time stamps, and semantic context into structured events; (2) hybrid DBN-GAT modeling for self-supervised event correlation computation using optimized loss functions and distance-density joint adjustment; and (3) integrating temporal features with semantic correlations to produce coherent event timelines.

#### Event Extraction and Presentation

Event extraction and representation consist of 2 main processes: the extraction of basic event elements and the generation of event summaries incorporating the basic event elements. Event information is extracted from older adults’ life stories. The structured event representations include event type, event trigger word, time, location, participant, and event summary, and the formal expression is: $e_{i}=\left(type,{ei}_{i},{tm}_{i},{loc}_{i},{pa}_{i},{em}_{i}\right)$(1)

#### Structured Event Extraction

The module achieves coreference resolution by using ALBERT (A Lite Bidirectional Encoder Representations from Transformers for Self-supervised Learning of Language Representations) embeddings with a 512-token sliding window for context management. Similarity between candidate expressions is measured via cosine distance, with a threshold of 0.8 established to determine coreferentiality [26,27]. We adopt a semisupervised open domain event extraction approach to expand event templates through manual annotation of triggers and types, enabling systematic extraction of basic event elements (type, trigger, time, location, and participants) for structured representation of older adults’ life stories [28].

#### Context-Aware Summary Generation

This module builds a dual-level encoder-decoder architecture. It uses ALBERT-BiLSTM for encoding and combines BiLSTM decoding with dynamic attention mechanisms [16]. The model prioritizes event-centric content by jointly analyzing word or sentence-level features alongside spatiotemporal and participant context. For event-level summarization, the system selects the top three most salient sentences using maximal marginal relevance based on ALBERT’s cross-attention scores. This ensures that the selected content maximizes coverage of the event trigger while minimizing information redundancy between sentences.

#### Event Relevance Calculation

##### Overview

After the event extraction module extracts the structured events for each life story, we need to calculate event correlations to arrange the event nodes on the timeline.

**Figure 2.**
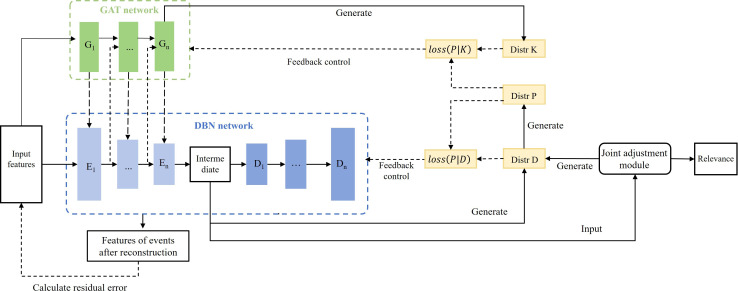
Detailed architecture of the event relevance computing model. This diagram illustrates the collaborative workflow between the graph attention network (GAT; which captures global dependencies) and the deep belief network (DBN; which performs feature compression). The joint adjustment module integrates the results to output refined correlation coefficients used for timeline sequencing.

The CARE-ET method uses a novel GAT-DBN collaborative model to compute event relationships for timeline construction [29]. The architecture combines: (1) an enhanced GAT network that captures multihop event dependencies through dynamic attention weights, extending influence beyond immediate neighbors; (2) a DBN component that learns compressed event representations while preserving relational semantics; and (3) a hybrid k-means or density-based spatial clustering of applications with noise (DBSCAN) self-supervision module that optimizes feature clustering through distance-density joint optimization [30], as shown in Figure 2. This integrated approach automatically learns hierarchical event relationships while maintaining computational efficiency through backpropagation-based training.

##### The Improved GAT Network

The proposed GAT extension structure advances traditional attention mechanisms by incorporating multihop node relationships during state updates. While standard GAT computes attention weights based solely on direct neighbors [31], our method dynamically integrates deeper network contexts through an adaptive depth strategy, enabling more comprehensive feature representation while maintaining computational efficiency. This enhancement allows each node to capture richer structural patterns by considering both immediate connections and semantically relevant indirect relationships within life story narratives.

**Figure 3.**
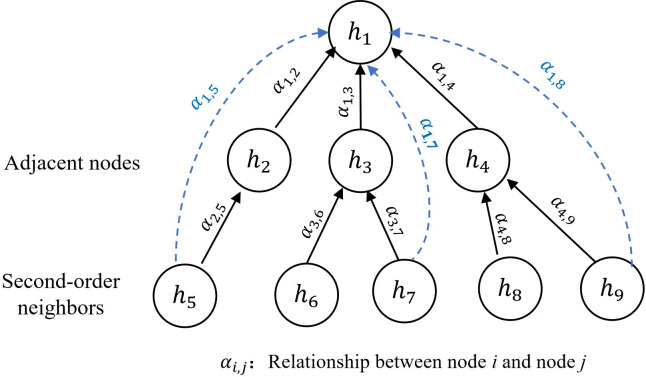
The mechanism of graph attention network node updates based on multihop relationships. Circles represent events ℎ_i_. Solid arrows indicate direct influence from immediate neighbors, while dashed arrows denote filtered indirect connections identified by the adaptive depth strategy. Gray nodes (eg, ℎ_6_ and ℎ_8_) represent connections that have been discarded due to the similarity threshold.

Figure 3 illustrates the GAT network’s enhanced state update mechanism, where each node’s output (eg, ℎ_1_) incorporates weighted influences from both direct neighbors (ℎ_2_, ℎ_3_, ℎ_4_) and secondary neighbors (ℎ_5_, ℎ_7_, ℎ_9_). The model automatically filters insignificant connections (eg, ℎ_6_ and ℎ_8_) through threshold-based attention weights, enabling efficient multihop relationship modeling while maintaining computational efficiency. This dynamic depth perception improves event relationship characterization without compromising network performance.

Assuming that a total of 𝑛 events exist, 𝑒 denotes the event, and each event is characterized as an 𝑚-dimensional vector. All event features can be represented as the set 𝛺 = {𝑒_1_,𝑒_2_,⋯,𝑒_𝑛_}. Based on this, the features of all events can be integrated into a sample feature matrix 𝛷 of 𝑛 × 𝑚:


(2) $$\begin{array}{c} \Phi =\left[\begin{array}{cccc} e_{11} & e_{21} & \cdots & e_{n1}\\ e_{12} & e_{22} & \cdots & e_{n2}\\ \vdots & \vdots & \cdots & \vdots \\ e_{1m} & e_{2m} & \cdots & e_{nm} \end{array}\right] \end{array}$$

The GAT network can be described as：


(3) $$\begin{array}{c} O\;=\;G\left(\Phi \right) \end{array}$$

The improved GAT network facilitates node feature evolution through linear transformations and attention-weighted aggregation. The feature update for each node in a specific layer is defined as:


(4) $$\begin{array}{c} h_{i}^{l}=\sigma \left(\sum_{j\in \left(N_{i}\cup I_{i}\right)}a_{ij}^{l}W^{l}\vec{h_{j}^{l}}\right) \end{array}$$

where 𝛷 is the network input, 𝒢 denotes the network function, and 𝒪 denotes the network output. Thus, the GAT network can be described as：


(5) $$\begin{array}{c} O=\left[\begin{array}{cccc} o_{11} & o_{21} & \cdots & o_{n1}\\ o_{12} & o_{22} & \cdots & o_{n2}\\ \vdots & \vdots & \cdots & \vdots \\ o_{1m^{\prime }} & o_{2m^{\prime }} & \cdots & o_{nm^{\prime }} \end{array}\right] \end{array}$$

where 𝑚′ is the new dimension of each event vector element, the actual output is a matrix consisting of new vectors describing each event.

The core of the GAT model lies in the graph attention layer, which maps the upper-layer graph node states to the lower-layer graph node states. The input to the graph attention layer, ℋ^l-1^, is the set of graph node feature vectors output from the upper layer.


(6) $$\begin{array}{c} H^{l-1}=\left\{e_{1}^{H^{l-1}},e_{2}^{H^{l-1}},\cdots ,e_{n}^{H^{l-1}}\right\} \end{array}$$

𝑛 is the number of nodes in the network, and 𝑓^𝑙−1^ is the dimension of the output features of the previous layer. Thus, the size of the matrix ℋ^l−1^ is 𝑛 × 𝑓^𝑙−1^:


(7) $$\begin{array}{c} H^{l-1}=\left[\begin{array}{cccc} h_{11}^{H^{l-1}} & h_{21}^{H^{l-1}} & \cdots & h_{n1}^{H^{l-1}}\\ h_{12}^{H^{l-1}} & h_{22}^{H^{l-1}} & \cdots & h_{n2}^{H^{l-1}}\\ \vdots & \vdots & \cdots & \vdots \\ h_{1f^{l-1}}^{H^{l-1}} & h_{2f^{l-1}}^{H^{l-1}} & \cdots & h_{nf^{l-1}}^{H^{l-1}} \end{array}\right] \end{array}$$

The output of this layer is a new set of node feature vectors $M^{\prime }$, denoted as：


(8) $$\begin{array}{c} M^{\prime }=\left\{\begin{array}{c} e_{1}^{H^{l}},e_{2}^{H^{l}},\cdots ,e_{n}^{H^{l}} \end{array}\right\} \end{array}$$

The attention coefficient $\theta_{ij}$ is computed using a single-layer feed-forward neural network, parameterized by a weight vector a, which operates on the concatenated features of the node pair:


(9) $$\begin{array}{c} \theta_{ij}=\vec{a}\left(W{\vec{h}}_{i},W{\vec{h}}_{j}\right) \end{array}$$

(10) $$\begin{array}{c} \theta_{ij}=LeakyReLu\left({\vec{a}}^{T}\left[W{\vec{h}}_{i}||W{\vec{h}}_{j}\right]\right) \end{array}$$

The proposed model extends traditional GAT attention computation through a dynamic coefficient function 𝛼 that incorporates both direct neighbors 𝑁_𝑖_ and validated indirect connections 𝐼_𝑖_, as shown in Figure 3. By enhancing the masked attention mechanism with SoftMax regularization, the system captures multi-hop dependencies while maintaining stable gradient propagation, significantly improving relationship modeling for life event networks compared to standard single-hop approaches.

This formula represents the attention coefficient of node *i* to node *j*, where α is not a constant but a node-dependent function, and *W* denotes the trainable weight parameters. The key innovation of our GAT enhancement lies in its refined attention allocation mechanism.

Unlike traditional GAT models, which compute attention solely over the direct neighbors of node *i*, our improved masked attention mechanism extends the scope of attention coefficient computation, enabling the exploration of indirect node relationships. Additionally, we introduce a SoftMax function to normalize attention scores across both direct neighbors and selected indirect nodes meeting specific criteria (equation 9). This modification enhances information propagation and dependency modeling between nodes:


(11) $$\begin{array}{c} \alpha_{ij}=softmax_{j}\left(\theta_{ij}\right)=\frac{\exp\left(\theta_{ij}\right)}{\sum_{\left(k\in N_{i}\cup I_{i}\right)}\;\exp\left(\theta_{ik}\right)} \end{array}$$

where 𝑁_𝑖_ denotes the set of adjacent nodes for node *i*, while 𝐼_𝑖_ represents the set of qualified indirect nodes for node *i*. According to the definition of 𝑁_𝑖_ and $I_{i}$, only events with a similarity exceeding a predefined threshold are established as related events.

Our GAT extension introduces an adaptive second-hop set $I_{i}$ based on semantic relevance thresholds. While heterogeneous graph attention typically focuses on predefined meta-paths [32], our approach dynamically identifies high-influence indirect neighbors in the narrative graph. By redefining the training of attention coefficients α over $I_{i}$, the model captures long-range dependencies in disorganized oral histories while mitigating the oversmoothing issues common in deep GNNs.

The network function 𝒢 determines the node influence weights based on the correlation between the 2 events. The cosine similarity function is used to calculate the event similarity 𝜑 between the 2 events 𝑒_𝑖_ and 𝑒𝑗:


(12) $$\begin{array}{c} \varphi \left(e_{i},e_{j}\right)=\frac{e_{i}\cdot e_{j}}{\left|e_{i}\right|\left|e_{j}\right|} \end{array}$$

To model the structural relevance of life events, we define the neighbor set $N_{i}$ for a given event node $e_{i}$ as:


(13) $$N_{i}=\{e_{j}|\text{cos}\text{\_}\text{sim}\left(h_{i},h_{j}\right)§amp;gt;\tau_{1}\}$$

where $\tau_{1}=0.6.$

For global consistency adjustment, the second-order inference set $I_{i}$ is constructed as:


(14) $$I_{i}=\{e_{k}|\exists e_{j}\in N_{i},\text{cos}\text{\_}\text{sim}\left(h_{j},h_{k}\right)§amp;gt;\tau_{2}\}$$

where $\tau_{2}=0.8.$

Next, the LeakyReLU function is adopted as the single-layer feed-forward function of the attention mechanism, and the improved attention mechanism is shown below：


(15) $$\begin{array}{c} \alpha_{ij}=softmax_{j}\left(\sigma_{ij}\right)=\frac{\exp\left(LeakyReLu\left({\vec{a}}^{T}\left[W{\vec{h}}_{i}||W{\vec{h}}_{j}\right]\right)\right)}{\sum_{\left(k\in N_{i}\cup I_{i}\right)}exp\left(LeakyReLu\left({\vec{a}}^{T}\left[W{\vec{h}}_{i}||W{\vec{h}}_{k}\right]\right)\right)} \end{array}$$

The attention function can calculate the attention coefficients between different nodes and predict the output characteristics of each node. The output characteristics are：


(16) $$\begin{array}{c} {\vec{h}}_{i}=\sigma \left(\sum_{j\in \left(N_{i}\cup I_{i}\right)}\;a_{ij}W{\vec{h}}_{j}\right) \end{array}$$

Meanwhile, this method adopts the multihead attention mechanism, which can make the attention training more stable. The core idea is to execute *m* attention functions independently and use the average distribution of *m* attention outputs as the output of the overall attention mechanism：


(17) $$\begin{array}{c} \vec{h_{i}^{\prime }}=\sigma \left(\frac{1}{m}\sum_{1}^{m}\;\sum_{j\in \left(N_{i}\cup I_{i}\right)}\;a_{ij}^{m}W^{m}{\vec{h}}_{j}\right) \end{array}$$

##### GAT Synergy With DBN Networks

The model integrates GAT and DBN through a bidirectional encoding-decoding framework. GAT captures interevent correlations via attention-weighted node interactions, while DBN performs hierarchical feature compression through multilayer transformations. Their synergistic operation—alternating GAT’s encoded outputs with DBN’s hidden states during each encoding round—preserves individual event characteristics while enhancing semantic relationships. The complementary decoding process ensures reconstructed features maintain fidelity to original inputs while optimizing them for correlation computation, achieving both dimensionality reduction and relationship-aware representation learning.

In this synergistic interaction model, the inputs and outputs of each layer of the DBN coding part are shown in equation 16：


(18) $$\begin{array}{c} h_{i}^{+}=sigmoid\left(\sum_{j}\;\left|H_{j}\right|w_{j}v_{j}+b_{i}\right) \end{array}$$

Also, the inputs and outputs of each GAT network can be adapted in the following form:


(19) $$\begin{array}{c} O_{i}=G\left(O_{i-1},h_{i-1}^{+}\right) \end{array}$$

##### Self-Monitoring Module

To address the coordination challenge between GAT and DBN networks, we introduce a novel self-supervised joint adjustment module that serves as an integrative optimizer. This module dynamically aligns the feature representations from both networks during prediction by: (1) generating target distributions through hybrid k-means or DBSCAN clustering, (2) constructing a unified loss function for backpropagation, and (3) enabling simultaneous parameter optimization across both architectures. The resulting feature representations are specifically tuned for accurate event correlation computation while maintaining the distinct advantages of each network’s processing paradigm.

If the 2 events are highly correlated, their features should also be highly similar. Multiple events describing similar contexts will form clusters in the feature space. In this paper, we use the output features of the DBN as inputs to the joint adjustment module to compute the interevent correlation and cluster structure. This is done by calculating the similarity between each event feature within a cluster and the center event feature of that cluster, which in turn generates a distribution *D* that contains the correlations of all the events, where ℎ_i_ denotes the DBN feature of the i^th^ event, 𝑐𝑗 is the center event of the j^th^ cluster, and ℎ_cj_ is the center event feature and 𝑣_𝑐_ is a fixed parameter. The construction of the correlation distribution is accomplished by traversing all clusters and their center events. The correlation distribution of the 𝑖^th^ event belonging to the 𝑗^th^ class is shown in equation 18:


(20) $$\begin{array}{c} d_{ij}=\frac{{\left(1+\frac{{\left|h_{i}-h_{cj}\right|}^{2}}{v_{c}}\right)}^{-\left(\nu_{c}+1\right)}}{\sum_{n}\;{\left(1+\frac{{\left|h_{i}-h_{cn}\right|}^{2}}{v_{c}}\right)}^{-\left(\nu_{c}+1\right)}} \end{array}$$

The self-supervised module enhances event feature representations by optimizing correlations between individual events and their cluster centroids, thereby improving semantic similarity grouping among related events. The target distribution of event relevance is first computed 𝑃：


(21) $$\begin{array}{c} p_{ij}=\frac{d_{ij}^{2}/f_{i}}{\sum_{j^{\prime }}d_{ij}^{2}/f_{i}} \end{array}$$

where 𝑓_𝑗_ = ∑_𝑖_ 𝑑_𝑖𝑗_ is the sum of the frequencies of a category of event correlation, and $\sum_{j^{`}}d_{ij}^{2}/f_{i}$ is the sum of the frequencies of all categories of event correlation, where 𝑗′ denotes the individual categories in the computation. The following loss function is constructed from the distributions P and D for inverse training the DBN. The self-supervision module uses the Kullback-Leibler divergence to quantify the discrepancy between the predictive and target distributions. The joint loss function is formally defined as:


(22) $$\begin{array}{c} L=KL\left(P|D\right)=\sum_{i}\;\sum_{j}\;p_{ij}\log\frac{p_{ij}}{d_{ij}} \end{array}$$

GAT also requires backpropagation training. Since both distributions P and D depend on the features generated by the DBN, more noise may be introduced if the loss(P|D) is used directly to compute the loss of GAT. The GAT network itself can generate a distribution 𝐾 (which is also the output of the last GAT network 𝒪), using K instead of the DBN-generated distribution D, using K and P to compute the loss. In this way, the GAT-generated distribution K is supervised by the distribution P, and the loss is designed to be used for the backpropagation of the GAT, where 𝑘_𝑖𝑗_ is 𝑜_𝑖𝑗_ in the last network output matrix 𝒪.


(23) $$\begin{array}{c} loss\left(P|K\right)=\sum_{i}\;\sum_{j}\;p_{ij}\log\frac{p_{ij}}{k_{ij}} \end{array}$$

Intuitively, the self-monitoring module acts as an “internal auditor.” While GAT makes initial predictions about event relevance based on local connections, the DBN provides a global thematic template. The module calculates the “surprise” or discrepancy between these two (via Kullback-Leibler divergence) and forces the model to self-correct, ensuring that local narrative links do not deviate from the overall life story logic.

The optimal number of clusters K in the joint adjustment module is determined using the silhouette coefficient and the elbow method applied to a validation subset of narratives. To prevent overfitting, we use L2 regularization and dropout (rate=0.5) within the GAT layers. Furthermore, the self-supervised loss function itself acts as a structural regularizer, penalizing the model when it focuses excessively on transient noise in individual narratives at the expense of global consistency.

The joint loss function is configured as $L_{total}=\lambda_{1}L_{GAT}+\lambda_{2}L_{joint}$, where $\lambda_{1}=0.7$ and $\lambda_{2}=0.3$ were empirically chosen to prioritize structural accuracy while maintaining global coherence. Optimization was performed using the AdamW routine with a weight decay of 0.01 and a linear learning rate scheduler that warmed up for the first 10% of total training steps.

**Figure 4.**
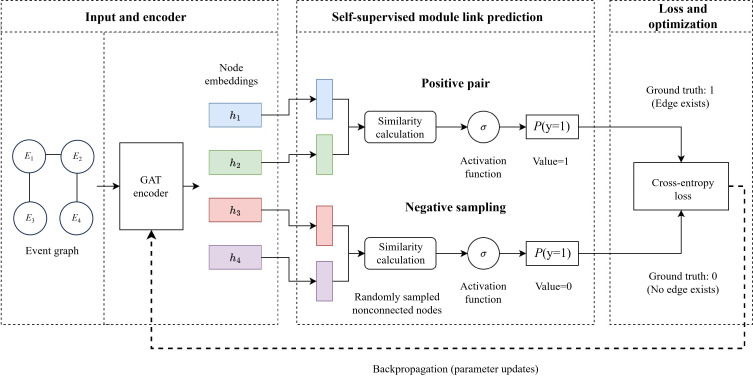
Self-supervised link prediction. A graph attention network (GAT) encoder generates node embeddings $h_{i}$ from the input graph. Positive and negative pairs are evaluated via dot products and sigmoid activation. The model minimizes cross-entropy loss through backpropagation to update encoder parameters and optimize latent representations.

Figure 4 illustrates the operational mechanism of the self-supervised module.

##### Joint Relevance Adjustment

**Figure 5.**
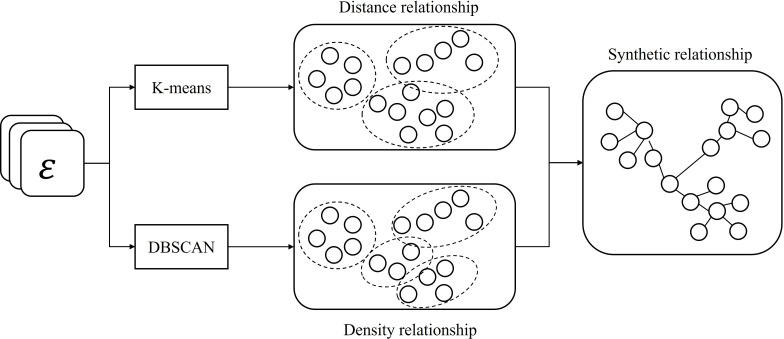
Framework of the joint adjustment module combining geometric and density metrics. The process integrates k-means (based on Euclidean distance to centroids) and density-based spatial clustering of applications with noise (DBSCAN; based on local density connectivity). The final correlation score is a weighted fusion of these 2 metrics to ensure structural robustness in narrative clustering.

The proposed method integrates k-means and DBSCAN clustering to compute event correlations through a dual-metric approach, as shown in Figure 5. K-means evaluates co-clustered events using relative centroid distances, while DBSCAN assesses connectivity through minimum path lengths between nodes. Both algorithms assign zero correlation to nonclustered event pairs.

The final correlation coefficient synergistically combines both clustering results, leveraging k-means’ geometric precision and DBSCAN’s density awareness. This hybrid approach captures complementary aspects of event relationships while maintaining computational efficiency through conditional zero assignments for unrelated events.

### Timeline Generation

To address temporal ambiguity in life stories, we first standardize diverse time expressions (eg, relative dates like “three years ago” or historical references like “Liberation War”). For events lacking absolute dates, we calculate cosine similarity between their text features and a curated historical event database, assigning standardized timestamps when similarity exceeds 0.85. This process ensures consistent temporal representation across both precise and ambiguous event references.

The generation algorithm first arranges temporally annotated events chronologically as the timeline backbone. For undated events, it calculates semantic similarity with existing timeline nodes to determine optimal insertion points between adjacent events. To integrate undated events $E_{u}$ into the anchor timeline $T_{anchor}=\{e_{1},e_{2},\ldots ,e_{n}\}$, we propose a relevance-based greedy insertion algorithm. For each $e_{u}\in E_{u}$, the optimal position i is determined by:


(21) $$\begin{array}{c} i^{*}=\arg\max_{0\leq i\leq n}\left(\alpha \cdot Rel\left(e_{i},e_{u}\right)+\left(1-\alpha \right)\cdot Rel\left(e_{u},e_{i+1}\right)\right) \end{array}$$

where $e_{0}$ and $e_{n+1}$ are virtual start and end nodes. If multiple positions yield the same maximum score, the tie is resolved by selecting the position that maintains the original narrative proximity in the transcript. This dual consideration of temporal sequence and semantic coherence produces timelines that maintain both chronological accuracy and narrative flow, essential for effective reminiscence therapy applications.

The cosine similarity threshold of 0.85 was empirically determined through a sensitivity analysis. In our pilot tests, thresholds above 0.95 resulted in a 36% loss of valid temporal anchors, while thresholds below 0.75 introduced significant “semantic drift,” where personal anecdotes were incorrectly mapped to unrelated historical events. To mitigate the impact of potential misalignment on timeline validity, we introduced a “confidence flag” mechanism: events with similarity scores between 0.70 and 0.85 are flagged for human-in-the-loop verification by caregivers. Error analysis indicates that inaccuracies primarily occur in narratives with vague temporal adverbs (eg, “back then”), which the system addresses by placing them in a relative sequence based on narrative flow rather than fixed historical coordinates.

The Historical Event Database used for mapping undated narratives contains approximately 5000 major historical events spanning from 1900 to 2000, curated from open-source repositories including Wikipedia and Baidu Baike. It focuses on events highly relevant to the Chinese older adult cohort (eg, social movements, economic reforms, and significant natural disasters). The database is maintained in Chinese and is licensed under CC-BY-SA 4.0 for research purposes.

### Story Mosaic System

The core architecture of the Story Mosaic system is built around three principal functional modules: (1) a story input module supporting multiple formats, including text, images, and videos, with temporal annotations available for manual or automatic addition; (2) a theme-based content organization module that allows for customizable categorization (eg, “Career” and “Family Life”); and (3) a chronological timeline generation module for key life events [17].

**Figure 6.**
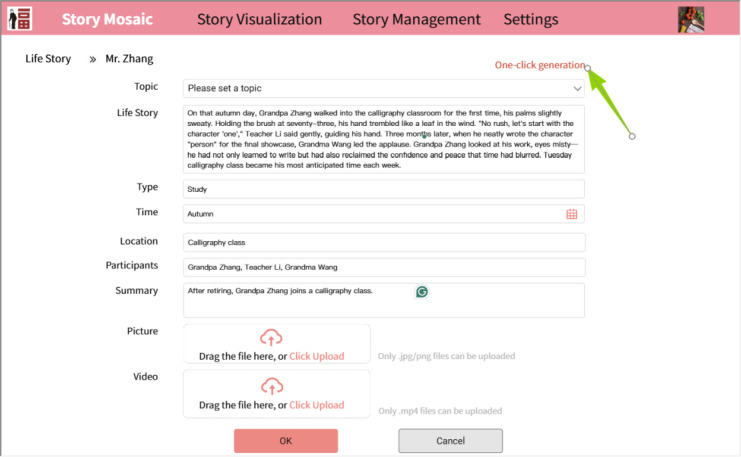
Schematic diagram of the automatic event feature generation module, supporting 1-click generation of event features.

In the preliminary work of the Story Mosaic system, the generation of life event timelines, while algorithmically automated, exhibited limited accuracy, with event elements largely reliant on manual annotation during story input. In this study, the timeline generation module has been specifically optimized based on the CARE-ET method. This enhanced module leverages the structured framework of the CARE-ET approach, integrating three core functions: event extraction, temporal sequencing, and narrative coherence, as shown in Figure 6. It enables the automated generation of accurate event summaries and timelines, thereby alleviating the data entry burden on caregivers.

## Results

We conducted a comprehensive evaluation of the proposed system, organized into two key aspects: (1) quantitative assessment of event representation and timeline quality, and (2) systematic usability testing.

### Datasets

We selected 2 datasets for the experiments to verify the effectiveness of the event timeline generation method. The first is the self-constructed OALS (Older Adults’ Life Stories) dataset, which contains the event data TimID (timeline identity) that has been annotated with timelines. The second is the Chinese news summary dataset LCSTS (Large Scale Chinese Short Text Summarization), organized by the Harbin Institute of Technology, which is constructed based on Sina microblog text. For detailed information about the datasets, please refer to Multimedia Appendix 1.

### Experimental Setup

Our model was implemented using PyTorch 1.12 (PyTorch Foundation) and trained on an NVIDIA Tesla T4 GPU (16GB VRAM). The ALBERT-base-v2 model was fine-tuned for 10 epochs with a learning rate of 2×10⁻⁵, a batch size of 16, and a weight decay of 0.01 using the AdamW optimizer. Early stopping was implemented with a patience of 3 epochs based on validation loss. Random seeds were fixed at 42 for reproducibility. The more detailed parameters are listed in Table 1. For the CARE-ET framework, the GAT neighbor selection threshold was set to 0.6, and the joint adjustment threshold to 0.8. An 80/10/10 split was used for training, validation, and testing. To facilitate reproducibility, the code is provided as supplementary material for review and will be made public upon acceptance. The more detailed parameters are listed in Table 2.

**Table 1.** Training hyperparameters.

Parameter	Value
Framework	PyTorch 1.12 (PyTorch Foundation)
Hardware	NVIDIA Tesla T4 GPU (16GB VRAM)
Base model	ALBERT^a^-base-v2
Fine-tuning epochs	10
Batch size	16
Learning rate	2.00×10⁻⁵
Weight decay	0.01 (AdamW)
Optimizer	AdamW
Dropout rate	0.5
Early stopping	Patience=3 epochs
Random seed	42
Dataset split	80% / 10% / 10% (Train/Val/Test)

a ALBERT: A Lite Bidirectional Encoder Representations from Transformers for Self-supervised Learning of Language Representations.

**Table 2.** CARE-ET (CARE event timeline) and timeline generation parameters.

Parameter	Symbol and description	Value
GAT^a^ neighbor selection threshold	$N_{i}$ (selection)	0.6
Joint adjustment threshold	$I_{i}$ (second-order neighbor)	0.8
Structural loss weight	$\lambda_{1}$ (structural accuracy)	0.7
Global coherence weight	$\lambda_{2}$ (consistency)	0.3
Temporal anchor similarity	Cosine threshold for history	0.85
Confidence flag range	For human-in-the-loop verification	[0.70, 0.85]
Coreference resolution window size	Sliding window for ALBERT^b^	512
Coreference resolution threshold	Cosine distance threshold	0.8

a GAT: graphic attention layer.

b ALBERT: A Lite Bidirectional Encoder Representations from Transformers for Self-supervised Learning of Language Representations.

The CARE-ET model was trained on a dataset of 3142 annotated narrative segments derived from 118 sessions. The annotation workflow involved 2 gerontology specialists who independently labeled event triggers and temporal relations; interannotator agreement reached a Cohen κ of 0.77. For model evaluation, we used a 5-fold cross-validation strategy to ensure the robustness of the results across different storytelling styles.

To ensure the statistical rigor of our results, we conducted bootstrap significance testing (n=1000) and used the paired 2-tailed *t* test to compare CARE-ET against the strongest baseline. Statistical significance is *P*<.05.

### Event Summary Assessment

Life story event summarization enables caregivers to efficiently retrieve relevant narratives while providing the structural foundation for organizing fragmented life stories, with evaluation conducted using Recall-Oriented Understudy for Gisting Evaluation (ROUGE) metrics [33]. ROUGE is a commonly used evaluation method for automated text summarization, which measures the quality of summaries by calculating the overlap between automated summaries and human-referenced summaries for unigram words (ROUGE-1), bigram words (ROUGE-2), and the longest common subsequence (ROUGE-L). The method is simple, intuitive, highly consistent with manual evaluation results, and is one of the most effective evaluation metrics for current text summarization tasks.

For automated summarization evaluation, we report the *F*_1_-scores of ROUGE-1, ROUGE-2, and ROUGE-L. All metrics were computed using the Python *rouge-score* package with Porter stemming enabled and standard English stopwords removed. To ensure the reliability of performance gains, we conducted bootstrap significance testing (n=1000) to report 95% CIs and *P* values against the baselines.

Table 3 and Table 4 present the experimental evaluations on both the OALS and LCSTS datasets [34], demonstrating CARE-ET’s superior performance in event summarization tasks compared to several baseline methods (evolutionary timeline summarization, Timeline-sumy, temporally sensitive submodularity framework for timeline summarization, crisis local timeline line-up summarization, pretraining with extracted gap-sentences for abstractive summarization, LongT5, and pyramid-based masked sentence pretraining for multidocument summarization) [23-25,35-38]. Using median and mean text lengths as input parameters (58/210 bytes for OALS; 234/248 bytes for LCSTS), CARE-ET consistently achieved higher accuracy across all test conditions, with particularly notable advantages in full-length input scenarios, showing 0.6% (SD 1.20%) and 1.6% (SD 0.69%) average improvements over the strongest baseline in OALS and LCSTS, respectively. These results validate the model’s effectiveness in extracting structured event summaries from both domain-specific life story data (OALS) and general narrative texts (LCSTS), confirming its robust generalization capabilities while maintaining processing accuracy across variable input lengths.

**Table 3.** Performance on the OALS (Older Adults’ Life Stories) dataset using different lengths.

Method	Length limited within 60 bytes (%), mean (SD)			Length limited within 220 bytes (%), mean (SD)			Full length (%), mean (SD)		
	ROUGE-1^a^	ROUGE-2^b^	ROUGE-L^c^	ROUGE-1	ROUGE-2	ROUGE-L	ROUGE-1	ROUGE-2	ROUGE-L
ETS^d^	26.4 (1.15)	13.9 (0.72)	19.5 (0.98)	31.1 (1.28)	14.3 (0.65)	26.3 (1.05)	34.9 (1.42)	15.1 (0.74)	30.1 (1.25)
Timeline-Sumy	26.4 (1.08)	13.9 (0.68)	19.5 (0.91)	31.1 (1.21)	14.3 (0.61)	26.3 (0.98)	34.9 (1.35)	15.1 (0.71)	30.1 (1.18)
TSSF-TLS^e^	26.9 (1.02)	15.5 (0.58)	19.9 (0.85)	31.3 (1.15)	15.9 (0.55)	26.9 (0.92)	35.8 (1.28)	15.9 (0.65)	30.8 (1.12)
CrisisLTLSum^f^	27.5 (0.95)	16.9 (0.62)	20.9 (0.78)	32.1 (1.08)	17.7 (0.52)	27.5 (0.88)	37.2 (1.15)	16.9 (0.68)	31.6 (1.05)
PEGASUS^g^	27.3 (0.91)	17.2 (0.55)	21.5 (0.82)	32.5 (1.02)	18.1 (0.48)	27.9 (0.85)	37.4 (1.12)	17.2 (0.58)	31.9 (0.98)
LongT5^h^	27.8 (0.88)	17.1 (0.52)	21.3 (0.75)	32.4 (0.98)	17.9 (0.45)	28.1 (0.82)	37.7 (1.08)	17.1 (0.55)	32.1 (0.95)
PRIMERA^i^	28.1 (0.82)	17.1 (0.48)	21.7 (0.68)	32.6 (0.92)	17.9 (0.42)	28.2 (0.78)	37.6 (0.98)	17.9 (0.52)	32.6 (0.88)
CARE-ET^j^ (Word2Vec)	27.9 (0.85)	17.6 (0.51)	21.9 (0.72)	32.7 (0.95)	18.2 (0.45)	28.4 (0.81)	37.9 (1.02)	16.9 (0.55)	32.4 (0.91)
CARE-ET (LSTM^k^)	28.5 (0.78)	17.7 (0.45)	21.7 (0.65)	33.1 (0.88)	18.6 (0.38)	28.7 (0.75)	38.1 (0.95)	17.4 (0.48)	32.8 (0.85)
CARE-ET	28.3 (0.72)^l^	17.5 (0.42)^l^	22.1 (0.61)^l^	33.2 (0.85)^l^	18.8 (0.35)^l^	28.9 (0.72)^l^	38.5 (0.92)^l^	17.7 (0.45)^l^	33.2 (0.82)^l^

a ROUGE-1: Recall-Oriented Understudy for Gisting Evaluation, variant 1.

b ROUGE-2: Recall-Oriented Understudy for Gisting Evaluation, variant 2.

c ROUGE-L: Recall-Oriented Understudy for Gisting Evaluation–Longest Common Subsequence.

d ETS: evolutionary timeline summarization.

e TSSF-TLS: temporally sensitive submodularity framework for timelines summarization.

f CrisisLTLSum: crisis local timeline line-up summarization.

g PEGASUS: pretraining with extracted gap-sentences for abstractive summarization.

h LongT5: long text-to-text transfer transformer.

i PRIMERA: pyramid-based masked sentence pretraining for multidocument summarization.

j CARE-ET: CARE event timeline.

k LSTM: long short-term memory.

l Statistically significant, *P*<.05.

**Table 4.** Performance on the LCSTS (Large Scale Chinese Short Text Summarization) dataset using different lengths.

Method	Length limited within 234 bytes (%), mean (SD)			Length limited within 248 bytes (%), mean (SD)			Full length (%), mean (SD)		
	ROUGE-1^a^	ROUGE-2^b^	ROUGE-L^c^	ROUGE-1	ROUGE-2	ROUGE-L	ROUGE-1	ROUGE-2	ROUGE-L
ETS^d^	25.4 (0.72)	12.8 (0.35)	22.1 (0.52)	27.2 (0.78)	14.1 (0.38)	24.5 (0.61)	29.1 (0.82)	16.3 (0.45)	26.2 (0.68)
Timeline-Sumy	25.5 (0.68)	12.9 (0.32)	22.2 (0.48)	27.3 (0.74)	14.2 (0.35)	24.6 (0.55)	29.2 (0.78)	16.4 (0.42)	26.3 (0.65)
TSSF-TLS^e^	26.4 (0.65)	14.1 (0.28)	23.3 (0.45)	28.5 (0.71)	15.6 (0.32)	25.7 (0.52)	30.2 (0.75)	17.5 (0.41)	27.4 (0.62)
CrisisLTLSum^f^	29.1 (0.58)	16.5 (0.25)	26.2 (0.38)	31.4 (0.62)	18.2 (0.30)	28.3 (0.48)	33.1 (0.68)	20.2 (0.38)	30.1 (0.58)
PEGASUS^g^	34.2 (0.55)	21.3 (0.32)	31.1 (0.42)	36.5 (0.65)	23.1 (0.38)	33.2 (0.51)	38.1 (0.71)	24.9 (0.42)	34.8 (0.61)
LongT5^h^	34.6 (0.51)	21.6 (0.28)	31.5 (0.38)	36.8 (0.58)	23.4 (0.32)	33.7 (0.48)	38.5 (0.65)	25.2 (0.38)	35.3 (0.55)
PRIMERA^i^	34.5 (0.48)	21.8 (0.25)	31.2 (0.35)	36.7 (0.55)	23.6 (0.28)	33.5 (0.45)	38.4 (0.62)	25.4 (0.35)	35.1 (0.52)
CARE-ET^j^ (Word2Vec)	34.9 (0.45)	22.1 (0.22)	31.8 (0.32)	37.1 (0.52)	23.9 (0.25)	33.9 (0.42)	38.9 (0.58)	25.8 (0.32)	35.8 (0.48)
CARE-ET (LSTM^k^)	35.3 (0.42)	22.4 (0.21)	32.2 (0.28)	37.4 (0.48)	24.2 (0.22)	34.2 (0.38)	39.2 (0.55)	26.1 (0.31)	36.2 (0.45)
CARE-ET	35.7 (0.38)^l^	22.7 (0.18)^l^	32.8 (0.25)^l^	37.8 (0.42)^l^	24.5 (0.21)^l^	34.8 (0.35)^l^	39.5 (0.52)^l^	26.4 (0.28)^l^	36.9 (0.42)^l^

a ROUGE-1: Recall-Oriented Understudy for Gisting Evaluation, variant 1.

b ROUGE-2: Recall-Oriented Understudy for Gisting Evaluation, variant 2.

c ROUGE-L: Recall-Oriented Understudy for Gisting Evaluation–Longest Common Subsequence (ROUGE-L).

d ETS: evolutionary timeline summarization.

e TSSF-TLS: temporally sensitive submodularity framework for timeline summarization.

f CrisisLTLSum: crisis local timeline line-up summarization.

g PEGASUS: pretraining with extracted gap-sentences for abstractive summarization.

h LongT5: long text-to-text transfer transformer.

i PRIMERA: pyramid-based masked sentence pretraining for multidocument summarization.

j CARE-ET: CARE event timeline.

k LSTM: long short-term memory.

l Statistically significant, *P*<.05.

To evaluate the specific contributions of each component, we conducted a rigorous ablation study. Table 5 indicates that replacing ALBERT with Word2Vec leads to a 1% drop in *F*_1_-score, highlighting the necessity of contextual embeddings. Furthermore, the GAT-DBN synergy outperforms GAT-only and DBN-only configurations by 2.8% and 3.4%, respectively. The self-supervised module provides an additional 0.9% gain in timeline consistency. Our model contains 64M parameters, and the average training time per fold is approximately 4.5 (SD 0.34) hours on a Tesla T4 GPU, representing a balanced tradeoff between complexity and performance.

**Table 5.** Ablation experiment results.

Configuration	ROUGE-1^a^	ROUGE-2^b^	ROUGE-L^c^	SLEU^d^
CARE-ET^e^ (Full)	*38.5* ^f^	*17.7*	*33.2*	*0.72*
Without ALBERT^g^ (Word2Vec)	37.4	16.8	32.2	0.69
Without BiLSTM^h^ (LSTM^i^)	37.3	16.5	32.0	0.68
Without DBN^j^ (GAT^k^ only)	35.7	15.2	30.4	0.61
Without GAT (DBN only)	35.1	14.8	29.8	0.58
Without self-supervised module	37.6	17.2	32.3	0.65
Without all (baseline Seq2Seq)	34.2	13.5	28.5	0.52

a ROUGE-1: Recall-Oriented Understudy for Gisting Evaluation, variant 1.

b ROUGE-2: Recall-Oriented Understudy for Gisting Evaluation, variant 2.

c ROUGE-L: Recall-Oriented Understudy for Gisting Evaluation–Longest Common Subsequence.

d SLEU: sequence link evaluation.

e CARE-ET: CARE event timeline.

f Italicized numbers indicate the best result among all experimental results.

g ALBERT: A Lite Bidirectional Encoder Representations from Transformers for Self-supervised Learning of Language Representations.

h BiLSTM: bidirectional long short-term memory.

i LSTM: long short-term memory.

j DBN: deep belief network.

k GAT: graphic attention layer.

### Qualitative Analysis

To further understand the performance of CARE-ET, we present 2 anonymized case studies comparing the generated timelines against the therapeutic ground truth.

#### Case 1 (Success in Long-Range Dependencies)

During an interview, the participant mentioned family-related milestones (eg, marriage and grandchildren) and their prime years in a later segment. CARE-ET effectively captured the cross-segment dependencies, chronologically positioning these events after related life stages while maintaining the semantic links between the memories.

#### Case 2 (Failure in Near-Duplicate Events)

During a session discussing a wedding, the participant mentioned the date and event details in different segments. The model failed to merge these, creating two independent nodes for the same event (a redundancy error). This failure suggests that current GAT-based relevance thresholding remains insufficient for fine-grained deduplication in repetitive, fragmented oral testimonies.

### Quality Assessment of Timelines

In this paper, sequence link evaluation (SLEU) metrics and TimID metrics are used to assess the quality of event timelines.

#### Sequence Link Evaluation

In this paper, the evaluation metrics of SLEU are used to verify the quality of the timeline [22]. The sentences in the system-generated storyline are 𝑠^1^, 𝑠^2^,… 𝑠^𝑛^, where *s*1 is the beginning of the story and 𝑠𝑛 is the end of the story. The sentences of the reference storyline are 𝑠^′^_1_, 𝑠^′^_2_, … 𝑠^′^_𝑛_. n is the number of stages, and h-gram and h-gram′ denote a sequence of h sentences in a storyline, not necessarily consecutive, generated by the reference storyline and the generated storyline. The collocation of information between sentences in a storyline is utilized to compute the probability of the occurrence of the storyline and thus determine whether the storyline is complete or coherent.

The cosine similarity between 𝑠^𝑖^(𝑖 ∈ [1,𝑛]) and 𝑠^𝑗^(𝑗 ∈ [1,𝑛]) is first calculated. Then the probability of h-gram matching between the 2 storylines 𝑃ℎ is calculated separately:


(22) $$\begin{array}{c} p_{h}=\frac{\min\left(Count_{match}\left(h-gram\right),Count_{match}\left(h-gram^{\prime }\right)\right)}{Count\left(h-gram\right)} \end{array}$$

The possible values of the variable h depend on the specific case and are represented by the set H. The set H is a set of 1-grams. Storylines using 1-grams tend to satisfy the synthesis requirement more easily. Longer h-gram matches indicate better coherence. The count (h-gram) denotes the number of comparable h-grams (eg, if h=4, count(2-gram)=6). The count(hgram’) equals count(h-gram). The count_match_(h-gram) denotes the number of h-grams that can be matched (ie, h-grams against h-grams). The count_match_(h-gram) denotes the number of h-grams that can be matched (ie, the number of h-gram pairs for which the similarity of the corresponding sentences exceeds a specific threshold λ). In the experiments, we set λ=0.2. Then SLEU =𝑒𝑥𝑝(∑ℎ∈𝐻 𝑤_ℎ_ log 𝑝_ℎ_) is computed. The SLEU metric value ranges from 0 to 1 and is used to quantitatively assess the quality of the storyline.

We standardized the timeline consistency metric as SLEU. It evaluates the structural integrity of the generated timeline by calculating the weighted sum of correct temporal transitions:


 (23) $$SLEU=\sum_{h=1}^{H}w_{h}\cdot \;exp\left(-\lambda \cdot \;\left|pos_{pred}-\;pos_{ref}\right|\right)$$

where H=3 denotes the maximum historical hop distance considered, $w_{h}$ represents the weight assigned to the h-th hop (set as 1/h), and λ=0.5 is the decay constant for distance penalties.

#### TimID

The study introduces TimID, a temporal annotation marker embedded within a novel life story dataset for older adults, enabling precise evaluation of event chronology through 2 complementary dimensions. Absolute position accuracy quantifies deviations between generated event nodes and their corresponding TimID reference points, where smaller positional differences indicate higher timeline fidelity. Simultaneously, relative order preservation assesses the correctness of pairwise event sequences by verifying their alignment with ground-truth temporal relationships. This dual-metric approach ensures comprehensive validation of both event placement precision and chronological logic in life story reconstruction.

TimID is defined to measure the precision of event placement. The “absolute position” of an event is defined as its normalized rank $r_{i}=i/N$, where N is the total number of events. The relative order preservation is computed using the Kendall τ coefficient, which quantifies the ordinal correlation between the predicted sequence and the gold-standard therapeutic timeline.

#### Experimental Results Based on SLEU and TimID

The study uses 2 complementary assessment frameworks: SLEU evaluates storyline coherence through sentence-level probability analysis, while TimID assesses timeline quality via absolute position accuracy and relative order consistency. As shown in Table 6, CARE-ET outperforms baseline methods across both metrics, achieving superior SLEU scores (0.021‐0.061 higher) and TimID performance (0.08%‐0.24%% better relative order accuracy, with 0.8‐1.7 lower positional deviations). These results demonstrate significant improvements in both narrative flow and temporal precision.

**Table 6.** Performance on the dataset OALS (Older Adults’ Life Stories) using sequence link evaluation (SLEU) and timeline identity (TimID).

Method	SLEU	TimID-ap^a^	TimID-rp^b^
ETS^c^	0.47 (0.02)	4.51 (0.38)	0.43 (0.03)
Timeline-Sumy	0.48 (0.02)	4.12 (0.25)	0.51 (0.03)
TSSF-TLS^d^	0.47 (0.02)	4.21 (0.31)	0.48 (0.03)
CrisisLTLSum^e^	0.51 (0.02)	3.64 (0.18)	0.59 (0.02)
CARE-ET^f^	0.53 (0.01)^g^	2.79 (0.15)^g^	0.67 (0.02)^g^

a TimID-ap: timeline identity–absolute position.

b TimID-rp: timeline identity–relatable position.

c ETS: evolutionary timeline summarization.

d TSSF-TLS: temporally sensitive submodularity framework for timeline summarization.

e CrisisLTLSum: crisis local timeline line-up summarization.

f CARE-ET: CARE event timeline.

g Statistically significant, *P*<.05.

The metrics validate CARE-ET’s dual capacity to generate clinically meaningful timelines: SLEU confirms enhanced narrative coherence, which is crucial for therapeutic applications, while TimID verifies improved chronological accuracy that aligns with human cognition. This combination of strong textual fluency and precise event sequencing enhances both the interpretability of life stories for caregivers and their therapeutic use in reminiscence interventions, advancing digital health solutions for older adult care.

### System Usability Assessment

This study has established cooperative relationships with the Hefei Tianyu Elderly Care Institution and the Chuzhou Lekang Elderly Care Institution. The system has been deployed within the daily care management platforms of both institutions, serving a total of 36 older adult users (21 at the Tianyu Institution and 15 at the Lekang Institution). For the optimized Story Mosaic system, we invited 4 caregivers and 1 manager from both the Tianyu and Lekang senior care facilities to complete the System Usability Scale (SUS) [39]. The statistical information for the 10 participants is shown in Table 7. Four participants (A1, C2, C3, and C4) had previously participated in the feasibility study of our published Story Mosaic system, while the other 6 were newly recruited for this study. The participants included 2 core roles—administrators and caregivers—with balanced gender representation. Aged 35 to 62 years, they represented young, middle-aged, and senior practitioner cohorts, while their elder care tenure (4‐23 y) reflected experiential disparities among novice, proficient, and senior workers. Their educational qualifications showed a gradient distribution, ranging from no formal education to master’s degrees. Participant selection ensured diversity across roles, sex, age, tenure, education, and affiliated institutions. This sampling strategy represented the target user groups and provided empirical support for the scientific rigor and objectivity of the system usability evaluation.

**Table 7.** Demographic characteristics of participants.

ID	Identity	Sex	Age (y)	Tenure	Education	Affiliated institution
A1	Administrator	Male	37	13	Master’s degree	Tianyu
A2	Administrator	Female	52	23	Junior high school	Lekang
C1	Caregiver	Female	56	10	Primary school	Tianyu
C2	Caregiver	Female	48	6	Junior high school	Tianyu
C3	Caregiver	Male	36	5	Junior high school	Tianyu
C4	Caregiver	Female	57	8	Primary school	Tianyu
C5	Caregiver	Male	35	4	Bachelor’s degree	Lekang
C6	Caregiver	Female	45	9	Junior high school	Lekang
C7	Caregiver	Female	62	14	Null^a^	Lekang
C8	Caregiver	Female	58	8	Primary school	Lekang

a Null: no education has been received.

**Figure 7.**
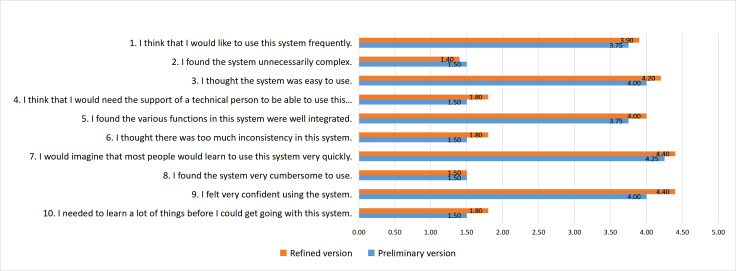
System Usability Scale scores from the preliminary and refined versions.

In the preliminary work of the Story Mosaic system, the average SUS score was 82.63, achieving an A– grade. After enhancing the system based on the CARE-ET algorithm, a usability test was conducted again, and the evaluation results are shown in Figure 7 with SUS scores from both the preliminary and refined versions.

A comparison of the refined and preliminary versions of the SUS scale shows the following: for positively worded items (1, 3, 5, 7, 9), the refined version scored higher, reflecting more positive evaluations of the system’s usability. For negatively worded items (2, 4, 6, 8, 10), the refined version still had relatively high scores, indicating that users perceived some remaining operational complexity. This is mainly because the refined version’s evaluators included caregivers with primary education or no formal education. While these users gave high scores on item 7 (“Most people can learn to use this system quickly”), their actual onboarding speed was slower. Moving forward, it will be necessary to simplify processes and add basic guidance for this group to improve their efficiency.

In addition to the above questions, we also asked an open-ended question: “What other ways do you think the timelines can help you care for older adults”? One participant noted:

*Timelines are critical in the care of older adults with cognitive impairment. These older adults' behavioral and psychological symptoms are closely related to their past experiences. Timelines help us understand their experiences and act appropriately to the situation.*

### Objective Comparisons

To objectively evaluate the impact of the Story Mosaic system on cognitive load, we conducted a comparative study between the artificial intelligence–assisted workflow and a traditional manual workflow. We used the NASA Task Load Index, which assesses cognitive demand across 6 dimensions: mental demand, physical demand, temporal demand, performance, effort, and frustration [40]. The comparative evaluation results are shown in Table 8. Focusing on the 3 core dimensions of mental demand, temporal demand, and physical effort prioritized by participants, the Story Mosaic system achieved load reductions of 37.5%, 36.4%, and 42.9%, respectively, in these metrics. It precisely addressed users’ key needs by effectively alleviating cognitive pressure, time constraints, and energy input. Meanwhile, the system also optimized loads across other dimensions, cutting the overall workload by 37.4% and demonstrating excellent human-machine compatibility.

**Table 8.** NASA Task Load Index (NASA-TLX) evaluation of the manual workflow and the Story Mosaic system.

Metric	Manual workflow, mean (SD)		Story Mosaic system, mean (SD)		Relative reduction (%)
Mental demand	76.0 (13.1)		47.5 (23.6)		37.5
Physical demand	27.0 (7.50)		25.0 (8.5)		7.4
Temporal demand	77.0 (10.1)		49.0 (19.3)		36.4
Performance	43.0 (16.7)		31.0 (16.3)		27.9
Effort	73.5 (13.8)		42.0 (15.1)		42.9
Frustration	62.5 (14.4)		34.0 (14.3)		45.6
Overall workload	65.7 (13.6)		41.2 (16.9)		37.4

## Discussion

### Principal Results

The GAT framework incorporates dynamic node filtering using multilevel semantic similarity thresholds, automatically preserving clinically meaningful life event relationships. This adaptive graph optimization enhances event relationship modeling precision while maintaining computational efficiency for real-world care applications.

CARE-ET demonstrates robust performance across both specialized life story datasets and general narratives through its novel integration of event triggers, spatiotemporal markers, and contextual encoding. The system outperforms conventional methods in extracting key life events from older adult narratives, providing a solid foundation for timeline construction.

Rigorous evaluation confirms that the generated timelines meet clinical standards for both narrative coherence and temporal accuracy. Caregiver assessments highlight superior readability and content generalization, validating the system’s ability to transform fragmented life stories into structured therapeutic materials for cognitive reminiscence therapy.

### Limitations

While CARE-ET demonstrates promising results, several limitations warrant consideration. First, the method’s performance relies heavily on the quality of temporal annotations (TimID) in the training data, which may limit generalizability to life stories with sparse or ambiguous time references. Second, the current implementation focuses primarily on textual narratives, potentially underutilizing multimodal cues (eg, emotional valence in vocal tones or visual context) that could enhance event interpretation. Third, the evaluation involved relatively small-scale caregiver assessments (n=10), necessitating broader clinical validation to confirm real-world utility across diverse older adult populations and care settings. Furthermore, the collection and processing of older adult individuals’ personal life stories involve highly sensitive data with significant privacy implications. Although this study followed ethical protocols including approval, informed consent, and data anonymization, several challenges persist. These include ensuring long-term data security, preserving participants’ ongoing control over their narratives, and addressing potential emotional impacts from recalling personal memories—each representing key ethical considerations in this research domain.

Addressing these current limitations, future work will focus on advancing research in the following areas: First, the development of weakly supervised temporal annotation enhancement algorithms will leverage cross-modal temporal reasoning and the mining of implicit temporal cues to improve the model’s robustness in parsing life stories with sparse temporal information. Second, the construction of a multimodal fusion framework that integrates speech emotion analysis, visual scene understanding, and textual semantic representation to establish a cross-modally aligned paradigm for life event comprehension. Third, collaboration with more elder care institutions to evaluate the long-term use of the system in real-world care scenarios.

### Conclusions

This study proposes the CARE-ET method, which enables the structured extraction of core events from life stories through multimodal event element fusion–based summary generation techniques and improves the accuracy of event relevance calculation via a collaborative computation model of GAT and DBN. Experiments on the OALS and LCSTS datasets show that CARE-ET significantly reduces redundancy and disorder in narrative texts, generating event timelines that outperform baseline methods in readability and structural rationality. This study provides an effective technical solution for the digital presentation of older adult life stories, with important application value for enhancing the personalization and precision of older adult care services.

## Supplementary material

10.2196/83122 Multimedia Appendix 1 Ethical approval of the dataset, data sources, construction basis, construction process, sample size, recruitment criteria, annotation specifications and processes for event elements and timelines, dataset public statement, etc.

## Abbreviations

ALBERT: A Lite Bidirectional Encoder Representations from Transformers for Self-supervised Learning of Language Representations
BiLSTM: bidirectional long short-term memory
CARE-ET: CARE event timeline
DBN: deep belief network
DBSCAN: density-based spatial clustering of applications with noise
GAT: graph attention network
LCSTS: Large Scale Chinese Short Text Summarization
OALS: Older Adults' Life Stories
ROUGE: Recall-Oriented Understudy for Gisting Evaluation
SLEU: sequence link evaluation
SUS: System Usability Scale
