# Supplementary material for: AI-Enhanced Automatic Life Story Structuring for Reminiscence Therapy in Older Adults: Technical Feasibility Study
Source: JMIR Aging. 2026 Apr 6;9:e83122. doi: 10.2196/83122 (PMC13052383; doi:10.2196/83122)
Supplement: Multimedia Appendix 1 [file aging-v9-e83122-s001.docx]

# Part One．Introduction to the OALS Dataset

The Older Adults' Life Stories dataset (OALS) contains 13,868 life story entries from 195 older adults. Based on this dataset, automated organization tasks such as event extraction, event summarization extraction, and timeline construction for older adults' life stories can be studied and validated.

Guided by the dataset construction documentation 'Datasheets for Datasets', our research team collaborated with multiple elderly care service institutions such as nursing homes and community centers to construct the OALS dataset of older adults' life stories. The dataset has been made publicly available on GitHub. It includes the following types of annotated data:

(1) Event element annotation, including event triggers, time, location, participants, and event summaries.

(2) Timeline annotation.

The dataset construction primarily involved four steps. The first step was collecting life story data, the second was data processing, the third was dataset expansion, and the fourth was data annotation. The construction process is illustrated in Figure 1.

Figure 1. The construction process of the OALS dataset

## 1. Collecting Life Stories

Collecting the life stories of older adults focuses first on designing an interview outline centered on life trajectories and key experiences, followed by uncovering authentic stories through face-to-face communication in field interviews.

### 1.1 Designing the Interview Outline

Recalling the past is not easy, and older adults often do not know where to begin. Therefore, before conducting interviews, it is necessary to scientifically design an interview outline. Relying on the guiding questions in the outline helps to establish a narrative framework for the interviewee, assisting older adults in anchoring their recollections and systematically organizing and presenting their life stories.

Under the guidance of Professor Pan Tianshu from the Department of Sociology at Fudan University and Professor Chen Honglin from the Department of Anthropology at the University of Eastern Finland, this study designed two complementary interview approaches: using key events as clues and using individual life cycles as clues. The event-clue module primarily focuses on areas such as marriage, work experience, educational background, and significant personal events of the older adults. The time-clue module uses life cycle stages as a framework, including childhood (6-12 years old), adolescence (12-18 years old), youth (18-40 years old), middle age (40-65 years old), and old age (65 years old and above).

To test and improve the rationality of the interview outline design, this study first conducted a pilot survey at the Hefei Jing'an Yangqinyuan nursing home, inviting ten older adults aged 65 and above to participate in life story interviews. After the pilot survey, the interview materials were systematically organized and analyzed. The results showed that when recalling life experiences, older adults not only used personal events and chronological order as main clues but also often relied on major national historical events to evoke related experiences and emotions, such as the period of the War of Resistance Against Japan, the Liberation War, the early years after the founding of the People's Republic of China, and the period since the reform and opening up. Based on these findings, the original interview outline was revised and supplemented accordingly, ultimately forming the interview outline shown in Figure 2, mainly consisting of three parts: event clue guidance, timeline clue guidance, and time clue guidance.

Figure 2. Life Story Interview Outline

### 1.2 Conducting Field Interviews

To conduct interviews with older adults more effectively within the nursing home context and systematically collect complete and rich life stories, our research team established partnerships with Hefei Jing'an Yangqinyuan, Hefei Tianyu Elderly Care Service Center, Shanghai Waitan Senior Service Center, and Shanghai Laoximen Senior Service Center. We invited older adults aged 65 and above with rich life experiences to participate in the study. Guided by the previously developed interview outline, researchers prompted respondents to narrate their life experiences from childhood to the present, including career journeys, family life, personal achievements, and challenges, to ensure the diversity and completeness of the life story data.

During the specific implementation, the study adopted a multiple, continuous interview strategy, conducting at least four to five in-depth interviews with the same respondent to more comprehensively capture their complex and multidimensional life experiences. The continuous interview process not only helped obtain more detailed information but also facilitated the establishment of a stable interviewer-interviewee relationship, encouraging respondents to share their life stories more fully. To ensure the accuracy and authenticity of the interview data, recording equipment was used throughout the interviews. Before the interviews, the purpose and use of the recording were fully explained to the respondents to obtain their understanding and consent.

This study has passed the ethical review of the Hefei University of Technology Research Ethics Committee (No.: HFUT20220921001) and the Chuzhou University Research Ethics Committee (No.: CZSC2025-021). All participants signed informed consent forms. Personal information and images mentioned in the text have been de-identified to effectively protect the privacy and sensitive information of older adults.

## 2. Data Processing

The goal of life story data processing is to convert the audio recordings of older adults' oral life stories collected during data acquisition into text, and to correct, clean, and organize the textual data. The objective is to compile each fragmented oral life story into a more readable text, with each life story saved as a separate text document. The process is as follows:

### 2.1 Audio Transcription

This study employed Automatic Speech Recognition (ASR) technology to transcribe the interview recordings into text, specifically using the iFlytek Hearing automatic speech recognition system. iFlytek Hearing is an intelligent speech transcription service launched by iFlytek. Based on mature speech recognition algorithms, it can efficiently and accurately convert speech to text. The system has strong technical advantages in Chinese and various dialect speech recognition, with systematic optimizations for different dialect characteristics. Therefore, it demonstrated high accuracy and adaptability in transcribing the oral life stories of older adults.

### 2.2 Data Verification

Given potential variations in accents or unclear pronunciation during interviews with older adults, automatic speech recognition results inevitably contain some errors. Therefore, after completing the automatic transcription, manual review of the text was conducted, comparing it with the original recordings one by one to promptly correct recognition errors. This ensures that the transcribed text matches the actual interview content, minimizing information bias and distortion as much as possible.

### 2.3 Data Cleaning

During text organization, temporal expressions mentioned in the interviews were standardized. Based on the specific time of the interview, relative time references like "this year," "last year," "a few months ago" were converted into explicit absolute times to enhance the accuracy of temporal information in the life stories. For colloquial expressions with distinct regional characteristics, place names and social titles involved were unified and standardized into more formal, commonly used expressions.

Simultaneously, strict privacy protection measures were applied to the text. Sensitive information such as personal identities, contact details, and specific addresses were deleted or replaced. Content potentially touching upon the privacy of the elderly or unsuitable for public disclosure underwent necessary redaction to avoid unnecessary distress or controversy, ensuring the compliance and ethical safety of the research data.

### 2.4 Data Confirmation

Each fragmented life story was saved as an independent document and submitted to the respective elderly respondent for review and confirmation. After re-soliciting their consent, a total of 16 older adults agreed to have their life stories publicly displayed.

## 3. Dataset Expansion

To expand the Older Adults' Life Stories dataset, this study collected publicly available information from the internet for 179 individuals aged 65 and above. The subjects covered various fields including generals, teachers, scientists, and doctors. Priority was given to individuals with relatively complete and information-rich life experience records online. Their names were used as keywords to search for related life story texts. To address noise in the web-sourced data, systematic cleaning was performed on the obtained texts: first, texts were segmented by paragraphs, and content under 20 characters was removed; secondly, based on common characteristic characters found in advertising text (such as "‖", "&", etc.), advertisement information was filtered out; finally, the initial 15,883 texts for the 179 individuals were manually reviewed to remove irrelevant or duplicate content, ultimately retaining 13,603 valid life story entries.

## 4. Data Annotation

### 4.1 Event Annotation

The Automatic Content Extraction (ACE) evaluation conference defines an event as an action, activity, occurrence, or situation of practical significance that happens at a specific time and place, which can be represented by keywords, phrases, and sentences in the text. An event typically includes the following elements:

(1) Event Type: Describes the basic category or type of the event, such as "Marriage," "Career," "Health," etc.

(2) Event Trigger: The word that triggers the event, usually a vocabulary item in the text that explicitly indicates the occurrence of the event, such as "served as," "fell ill," etc.

(3) Event Participants: Individuals related to the event, which can be people, organizations, etc.

(4) Event Time: The specific point in time or time range when the event occurred, which can be a date, time, or period, such as "August 1, 2023," "10:00 AM," etc.

(5) Event Location: The specific location where the event occurred, which can be a country, city, building, etc., for example, "Anhui," "company," etc.

(6) Event Attributes: Additional information describing the event, such as the cause, process, result of the event, etc.

This study conducted event element annotation on the life stories of older adults, categorizing the event types involved in the stories into six classes: Marriage, Education, Career, Family & Friends, Hobbies & Interests, and Others. The core event summary within each life story was used to describe and annotate the corresponding event attributes.

The annotation for each life story was completed jointly by three team members. During the data annotation phase, first, two annotators independently annotated all life stories to ensure data accuracy and consistency. Subsequently, a third annotator verified the results of the first two. When inconsistencies arose between the two annotators' results, they engaged in collective discussion to jointly determine the final annotation. This annotation method effectively reduced the impact of individual annotator subjectivity on data labeling, ensuring the stability and reliability of data annotation quality.

Figure 3 shows a data example from the dataset—a life story with annotated event elements and an event summary. Among them, the *person_id* represents the unique identity code of the older adult. In the dataset, each life story entry exists independently; life stories with the same *person_id* represent the life experiences of the same older adult. The *source_text* represents the original text of the life story. The *type* represents the event type within the life story. The *ei* represents the trigger word of the core event, which is an important marker for identifying the event. The *tm* represents the time when the core event in the story occurred. The *loc* represents the location where the core event in the story occurred. *pc* represents the participants in the core event of the story. The *em* represents the event summary text of the story, which is the descriptive information of the core event in the story.

Figure 3. Example of Annotation Event in a Life Story

### 4.2 Timeline Annotation

A timeline refers to the extraction and summarization of an older adult's life story, organizing a series of events in chronological order to generate a concise and meaningful timeline. In the context of older adults, constructing a timeline means organizing and summarizing their life stories, experiences, and events according to a chronological sequence to produce a concise yet representative timeline.

Timeline annotation was conducted based on the event annotation results of the life stories. During the event annotation process, event elements in each life story were identified and annotated, including temporal information. However, some events lacked explicit temporal elements. When constructing the timeline, event summaries with temporal elements were first sorted chronologically, and a unique timeline identifier (timeID) was assigned to each temporal node, using sequential numbering like "1, 2, 3, ..., n." For example, if the first event in an older adult's life story was "Grandpa Wu was born on September 1, 1920, in Changzhou City, Jiangsu Province," the timeline node corresponding to this event would be marked as timeID = 1.

For events lacking explicit temporal elements, processing was done by combining semantic relevance and historical era context. Specifically, by analyzing the content semantics and era characteristics of the event in relation to the nodes in the already sorted timeline, the event was inserted into the most reasonable position within the timeline, thereby ensuring the continuity and logical consistency of the overall timeline.

# Part Two. Introduction to the LCSTS Dataset

The LCSTS (Large Scale Chinese Short Text Summarization Dataset) is a large-scale Chinese short text summarization dataset. It aims to address the scarcity of high-quality, large-scale datasets in automatic text summarization research, providing standardized training and testing resources for Chinese short text summarization tasks. The dataset originates from Chinese Sina Weibo, with each data point presented in a "short text - summary" format. It includes 2.4 million training data entries, sufficiently supporting the training needs of deep learning models. The dataset has been made publicly available (http://icrc.hitsz.edu.cn/Article/show/139.html) for free academic use without requiring additional authorization.
